# Calculating the chemical exergy of materials

**DOI:** 10.1111/jiec.13120

**Published:** 2021-03-31

**Authors:** Charalampos Michalakakis, Jeremy Fouillou, Richard C. Lupton, Ana Gonzalez Hernandez, Jonathan M. Cullen

**Affiliations:** 1 Department of Engineering, University of Cambridge, Trumpington Street, Cambridge, CB2 1PZ UK; 2 Department of Mechanical Engineering, Centre for Sustainable and Circular Technologies (CSCT), University of Bath, Bath, UK; 3 Emerson, Madrid, Spain

**Keywords:** exergy, exergy calculation, industrial ecology, material efficiency, resource flows, software

## Abstract

Modern society requires large amounts of materials which lead to emissions of greenhouse gases. Effective climate policy should focus on not just energy efficiency but material efficiency as well. Exergy analysis is a powerful metric used to identify opportunities for efficiency improvement in industrial resource flow systems. Exergy offers a single unified measure of energy and material resources and indicates the real thermodynamic value of these resources, but the method suffers from a lack of comprehensive specific material chemical exergy datasets. The variety of different materials used in the global resource supply chain necessitates a combination of exergy calculation approaches. These approaches are combined into a single exergy calculator tool with over 1400 substances in the dataset. The chemical exergy values computed for key materials typically differ by less than 10% from values estimated in literature. The calculator is used in a case study of the upstream global material supply chain in 2013. The exergy resource map is visualized in a Sankey diagram and is found to be 72% resource efficient with 170 EJ of combined exergy losses and destruction. Further analysis is conducted on the refining, utilities, and industry sectors which are found to be 72%, 44%, and 50% resource efficient, respectively. Their combined losses and destruction are 15, 101, and 54 EJ, respectively. This study and the calculator developed provide a comprehensive dataset of chemical exergy values for a wide range of materials and can be applied in a variety of studies using exergy analysis.

**Supplementary Information:**

The online version of this article (doi:10.1111/jiec.13120) contains supplementary material, which is available to authorized users.

## INTRODUCTION

Levels of resource consumption and material demand have been rising steadily and are projected to increase, driven by economic growth (OECD, 2018). Because these materials are often energy‐intensive, their production has an adverse environmental effect as a result of the associated greenhouse gas (GHG) emissions (Lechtenböhmer et al., 2016). To reduce emissions, therefore, it is imperative to focus on both energy and material efficiency options (Allwood et al., 2010; Material Economics, 2019).

Reducing demand for materials and reducing supply chain inefficiencies are key strategies for emission mitigation; there is a need to identify losses within highly complex global material supply chains. Exergy is commonly used as a metric to analyze energy resource flows and can be defined as the “the amount of work obtainable for a system as it comes into a state of thermodynamic equilibrium with the surroundings through reversible processes” (Ahmadi et al., 2011). It acts as a quality index that represents the intrinsic value of a material or energy resource and combines those resources into a single metric of useful work. As a result, exergy provides insight into the relative magnitude of different types of resource flow.

When analyzing complex system flows with multiple streams and processes, such as global energy and material supply chains, Sankey and Grassmann diagrams can be used to visualize energy/materials and exergy flows, respectively. Sankey diagrams represent flows as lines or arrows whose width is proportional to the magnitude of the flows (Lupton & Allwood, 2017). Grassmann diagrams are similar but differ only in that they show exergy destruction as a reduction in thickness of flows across a particular process, instead of as a separate flow (Kotas, 1985). Combining these diagrams with energy and material flows expressed in exergy can allow for an illuminating and information‐dense representation of the complex interactions between these resources.

### Exergy as a metric

Using exergy as the metric for quantifying global flows of resources offers three key advantages. Firstly, it combines energy and materials into a single resource flow with common units, recognizing the complex interactions that these materials exhibit with each other (Gonzalez Hernandez et al., 2018). Secondly, contrary to energy and mass, exergy is not conserved and can be destroyed due to irreversible processes; this indicates potential for thermodynamic improvement in a system (Szargut, 2005). Finally, using the Second Law of thermodynamics (exergy) efficiency definition as opposed to a First Law (energy) efficiency helps quantify the usefulness or quality of the resource inputs and outputs, which is more representative of their real thermodynamic value (Koroneos et al., 2012).

However, a prominent drawback of exergy as a metric is the current lack of "specific exergy" values (exergy per kg or J of resource) for materials resources. Exergy values for some common resources are available, tabulated in the appendices of academic exergy textbooks. Nevertheless, a comprehensive database of specific exergy for different materials and energy carriers does not yet exist. Exergy data is particularly scarce for materials, where complex mixtures of elements make the calculation of exergy more challenging. Indeed, previous studies have identified the need for simpler exergy implementation guides and software (Gonzalez Hernandez & Cullen, 2019).

Databases of energy and material flows are widespread. International agencies publish detailed records of worldwide resource flows: for example, energy flows (IEA, 2015c) and aluminium flows (International Aluminium Institute, 2016). Conversely, there is no available database for global flows of exergy, and although there exist several exergy calculators usable online, these only return the specific exergy of a narrow range of chemical substances (Valero & Valero, 2017) without addressing more complex materials and energy carriers. Moreover, although some collections of data exist for a narrow range of substances (Szargut, 2005), no previous study has compiled these into a self‐contained and accessible database for use in research.

### Exergy components and chemical exergy

The thermodynamic concept of exergy underpins a number of approaches for quantifying resource use and loss. The exergy associated with a stream of material (assuming no nuclear, electromagnetic or surface tension effects, a common assumption of large‐scale systems analyses), can be divided into the following components: kinetic, potential, physical, and chemical exergy arising from the velocity, position, temperature, and pressure and the chemical composition of a material (Bejan et al., 1995; Kotas, 1985). The kinetic and potential components of exergy are typically neglected in industrial systems analysis (Nguyen et al., 2014). So, the physical and chemical components are the most important exergy components.

Physical exergy can be defined as: "*The maximum amount of work obtainable when the stream of substance is brought from its initial state to the environmental state defined by p*_*0*_* and T*_*0*_*, by physical processes involving only thermal interaction with the environment"* and chemical exergy as "*the maximum amount of work obtainable when the substance under consideration is brought from the environmental state to the dead state by processes involving heat transfer and exchange of substances only with the environment*" (Kotas, 1985). They both provide a measure of the useful energy that can be extracted from a substance due to its physical state and composition respectively and can be calculated based on known chemical properties. In order to accurately estimate how much exergy is entering and exiting a particular system, a good estimation of both of these components is necessary.

Physical exergy and the calculations required to estimate it are relatively straightforward with academic discussion on this topic moving to further disaggregate physical exergy into its pressure and temperature components (Morosuk & Tsatsaronis, 2019). Conversely, the calculations for chemical exergy are more complex with several different methods being developed for different material types. This complexity derives from various factors, including the need to define an appropriate reference environment for different substances (Abdollahi‐Demneh et al., 2011) and the complexity of bonds in different types of solid and liquid materials (Song et al., 2012). A more detailed outline of these different methods is given in Section 2.1.

### Exergy analysis of large‐scale systems

While exergy analysis has been traditionally used to investigate the thermodynamic performance of electricity generation equipment and plants, high oil prices in the 1970s prompted an increased interest in its application to large‐scale systems. Since then, studies have applied the concept of chemical EEA analysis at a national scale in countries such as Sweden, United States (Ayres et al., 2011), or China (Zhang et al., 2012). However, only few such studies, often limited to the national level (Soundararajan et al., 2014), produced a visual representation of exergy flows, with others preferring instead to produce a simple exergy efficiency measurement.

Other studies have attempted to derive estimates for regional and global resource efficiencies using exergy (Nakicenovic et al., 1996), but these relied on solving balance equations for selected control volumes. However, because of the use of balance equations instead of EEA, and the complexity of available data, this only estimated single average efficiency values, without representing exergy flows within the systems under consideration.

Specific industries have also been subjected to exergy analysis at a global scale, such as the cement industry (Boroumandjazi et al., 2013), or the steel industry (Gonzalez Hernandez et al., 2018). Sectoral‐level studies have also been undertaken at the national level: e.g. industrial sector in Mexico and Denmark (Bühler et al., 2016; Arango‐Miranda et al., 2018). These studies have mapped flows in their respective sectors yet a higher‐level approach encompassing interactions between those industries within the global supply chain can be more suitable for guiding high‐level policy.

A multi‐sector global analysis of industrial resource use has not been attempted using the exergy metric; only nation or sector‐specific studies exist. Part of the reason behind this is the lack of comprehensive chemical exergy data for the many material resources being used in industry.

This study’s aims are: firstly, to collate together in one place the relevant theory, calculation methods and data for calculating chemical exergy of a wide range of materials. Secondly, to describe the creation of a user‐friendly tool that builds on the previous point to provide the user chemical exergy with a distribution of results for these materials based on different methods. Thirdly, to demonstrate the application of this calculator tool and the relevant theory on a simplified global case study of energy and material flows. We obtain exergy efficiency and loss (combined exergy loss and destruction) values for the Utility and Refining sectors and five of the main industrial sectors namely: chemicals and petrochemicals, iron and steel, non‐metallic minerals, paper, and non‐ferrous metals.

This report follows this two‐staged approach: creation of the exergy calculator followed by its application in the creation of a global resource Sankey diagram. The design and implementation of the calculator along with the resource flow mapping approach are detailed in the next section. This is followed by a presentation of our results along with a discussion of their significance. The paper ends with an outline of the most relevant conclusions drawn from this work.

## METHODS

This section details the methodology for calculating exergy for materials, the development of an exergy calculator, and the approach used in mapping global industrial energy and material flows.

### Calculating exergy

In calculating specific exergy values for materials, only the chemical exergy of the substance is considered, that is, the exergy content of a material due to its chemical composition. The physical exergy, which arises from a material’s temperature and pressure difference from the environment, is neglected in this study as the materials are assumed to be at the restricted ambient conditions of *T*_0_ = 298.15 °C and *P*_0_ = 1 atm. This is done mainly because collecting temperature and pressure data for such a wide scope is infeasible.

To calculate the specific chemical exergy of molecules, compounds, or elements, Szargut introduced the concepts of "reference species" and "reference reactions" (Szargut, 2005). The standard chemical exergy of a range of chemical species has already been calculated in previous studies and tabulated in the literature using this reference model (Szargut, 2005). As the method of reference species and reactions requires a large amount of chemical data, these standard chemical exergies can be collected directly in the database, rather than calculated from first principles.

For a known and homogenous mixture of species, the standard molar chemical exergy can be computed using the formula by Szargut (Szargut, 2005) shown in Table Table 1.

**TABLE 1:** Formulae used for determining exergy across different material types. γ in Equation (1) is the activity coefficient correcting for non‐ideal gases, assumed here to be 1, as is typical with analyses such as this. Equations (6) and (7) are only applicable to biomass

Function	Expression	Equation	Unit	Reference
User‐devised homogenous mixture	$\kern0.28em {b}_{\mathrm{ch}}=\sum \limits_i{y}_i{b}_{chi}\kern0.56em +R{T}_0\sum \limits_i{y}_i\ln \left(, *{y}_i\right)\kern0.56em$	1	kJ/mol	(Szargut, 2005)
Ultimate analysis data (C, H, O, N, S, Ash in mass fractions)	$\kern0.28em {b}_{\mathrm{ch}}=\kern0.28em 8177.79\kern0.28em C+\kern0.28em 5.25\kern0.28em N+27892.63\kern0.28em H+4364.33\kern0.28em S-3173.66\kern0.28em O$ – 298.15 * 0.17152 Ash + 0.15 * O * {7837.667 C + 33888.889 H – 4236.1 O + 3828.75 S}	2	kcal/kg	(Shieh & Fan, 1982)
	$\kern0.28em {b}_{\mathrm{ch}}=\kern0.28em 32904.076\kern0.28em C+\kern0.28em 2040.24\kern0.28em N+117714.337\kern0.28em H+16341.556\kern0.28em S-13405.192$ O – 298.15 * 3.003 Ash + 0.15 * O * {32833.33 C + 141865.08 [H – O / 8] + 19500 S}	3	kJ/kg	(Stepanov, 1995)
	$\kern0.28em {b}_{\mathrm{ch}}=\kern0.28em 36343.9\kern0.28em C+107563.3\kern0.28em H-8630.80\kern0.28em O+414.7\kern0.28em N+19079.8\kern0.28em S-21100\kern0.28em Ash$	4	kJ/kg	(Song et al., 2012)
	$\kern0.28em {b}_{\mathrm{ch}}=\kern0.28em 92008\kern0.28em \left(\frac{1}{3}\kern0.28em C+H+\frac{1}{8}\kern0.28em S\right)$	5	kJ/kg	(Qian et al., 2017)
Higher heating value (HHV in kJ/kg)	$\kern0.28em {b}_{\mathrm{ch}}=\kern0.28em 1.047\kern0.28em \mathrm{HHV}$	6	kJ/kg	(Song et al., 2011)
	$\kern0.28em {b}_{\mathrm{ch}}=\kern0.28em 0.978\kern0.28em \mathrm{HHV}+2124.118$	7	kJ/kg	(Huang et al., 2016)
Lower heating value (LHV in kJ/kg)	$\kern0.28em {b}_{\mathrm{ch}}=\kern0.28em \mathrm{LHV}$ (solid fuels)	8	kJ/kg	(Rant, 2006)
	$\kern0.28em {b}_{\mathrm{ch}}=\kern0.28em 0.975\kern0.28em \mathrm{LHV}$ (liquid fuels)	9	kJ/kg	(Rant, 2006)
	$\kern0.28em {b}_{\mathrm{ch}}=\kern0.28em 0.95\kern0.28em \mathrm{LHV}$ (gaseous fuels)	10	kJ/kg	(Rant, 2006)
Lower heating value and ultimate analysis data	$\kern0.28em {b}_{\mathrm{ch}}=\kern0.28em \mathrm{LHV}\ast \left\{1.047+0.0154\frac{H}{C}+0.0562\frac{O}{C}\kern0.28em +0.5904\frac{N}{C}\left(1-0.175\frac{H}{C}\right)\right\}$ (liquid fuels)	11	kJ/kg	(Szargut, 2005)
	$\kern0.28em {b}_{\mathrm{ch}}=\kern0.28em \mathrm{LHV}\ast \left\{1.0347+0.0140\frac{H}{C}+0.0968\frac{O}{C}\kern0.28em +0.0493\frac{N}{C}\right\}$ (solid fuels)	12	kJ/kg	(Szargut, 2005)

Organic materials, however, typically feature complex and unpredictable chemical structures, and their chemical exergy cannot be estimated using the formula for homogenous materials. To address this, several studies have extracted empirical correlations from experimental data to estimate the chemical exergy of a range of organic materials. Table Table 1 shows different correlations which can be used to compute exergy from either an ultimate analysis data (UA) or from heating values.

UA estimates the mass fraction of basic elements in an organic sample, such as carbon, sulfur, and ash. The Higher Heating Value (HHV) of organic materials, listed in J/kg, corresponds to the total amount of heat energy liberated when a sample is completely burnt, and products of combustion are cooled to environmental conditions. Water vapor contained in exhaust gases are therefore condensed in the process. For the Lower Heating Value (LHV) water vapor leaves the combustion without being condensed.

Such data is available from several studies, for example, (Eboh et al., 2016), which have investigated properties of organic samples. This data has been compiled to use as inputs in the estimation of chemical exergy for organic materials.

### Specification for the exergy database and calculator

**FIGURE 1:**
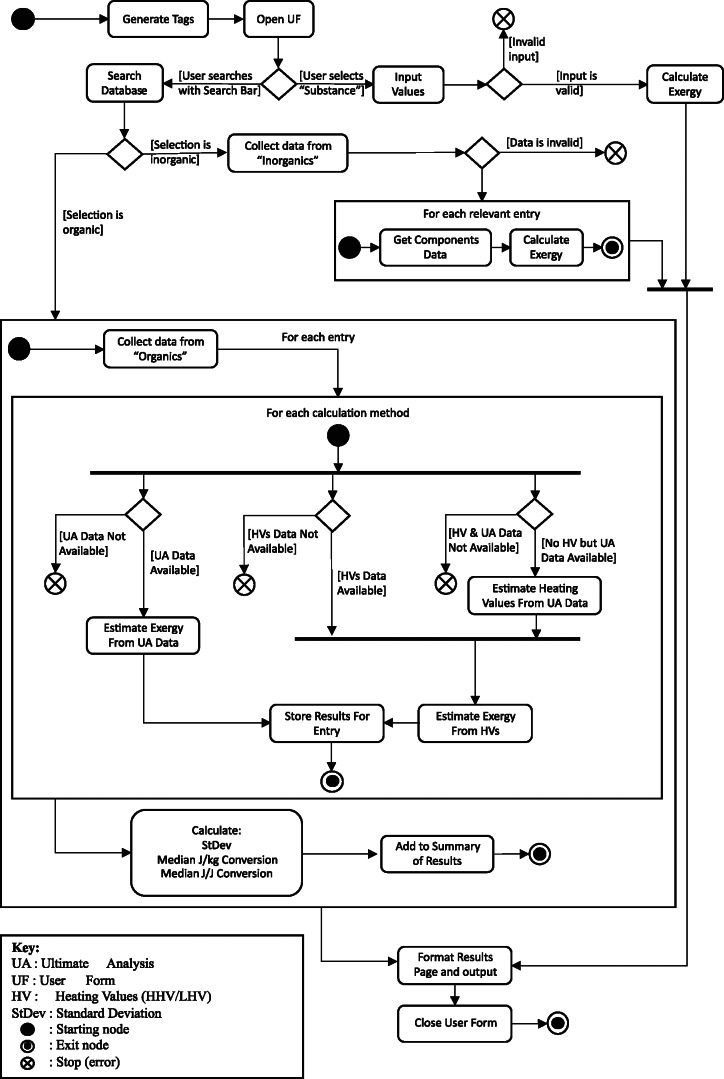
UML flow diagram of calculator operation for retrieving result

The ultimate aim of this research activity is to develop an “exergy calculator,” which returns a conversion factor for deriving the chemical exergy content for a wide range of substances. Therefore, a prototype exergy calculator was built in Microsoft Excel using VBA which allows the user to return an exergy value from a search query through a number of materials or from specific material composition data the user enters. The database allows tags to be assigned to materials; for example, bituminous coal might have the tags: "coal," "fuel," "fossil fuel," enabling the user to search more generally across a material grouping. The code written to convert a user input into an exergy value is summarized in the Unified Modeling Flow (UMF) process shown in Figure 1. The database is divided into three sections:

- A wide range of chemical exergy values for chemical species, calculated by other authors;
- Inorganic materials, such as steel, for which exact chemical compositions are given for different types (e.g. different steel alloys and grades);
- Organic materials such as biomass for which ultimate analysis data and heating values are used to calculate exergy.

The prototype calculator also features an option for inputting a customized substance, where up to eight chemical elements can be defined with their mass or atomic composition, based on which the substance’s specific exergy is calculated.

Building on the VBA implementation of the calculator, we also developed an online version^1^. This transfers all the calculations and methods outlined in this manuscript using the Django, Python, and HTML programming languages. It is publicly available to any exergy practitioner wishing to obtain chemical exergy values for a wide range of materials, more comprehensive than the materials featuring in the case study performed in this work.

The calculator returns results in the form of specific chemical exergy in units of kJ/mol and kJ/kg for substances and customized substances, a range of specific chemical exergies in kJ/kg for different versions of an inorganic material (e.g., glass) and for organic material, the median and standard deviation of a range of exergy values calculated by different empirical relationships is provided.

### Mapping global exergy flows

A map of exergy flows is developed to track material and energy resources through the global industrial system. This serves to demonstrate the usefulness of visualizing resource flows using exergy, and therefore the need for an exergy database and calculator. Data on the global flows of common materials and energy resources are widely available and summarized in Table 2. The raw data collected are available in Supporting Information S3. These values were matched with values in the exergy calculator, and then converted into Sankey diagram form using floWeaver, a Cambridge‐developed Python‐based tool (Lupton & Allwood, 2017). While Grassmann diagrams are traditionally used for visualizing exergy flows, exergy Sankey diagrams have become ubiquitous in recent years (Madlool et al., 2012; Soundararajan et al., 2014; Wu et al., 2016), as they are much more common in wider sustainability literature. In addition, the representation of exergy destruction as a "fictional flow" towards an exergy destruction node allows for easier comparison between the magnitudes of the exergy flows and exergy destruction from the different processes in the diagram.

**TABLE 2:** Definitions of material and energy groupings used in Sankey plotting

*Sector*	Info	Source
Chemicals:	Chemical and petrochemical sector including fertilizers, lubricants etc.	(Levi & Cullen, 2018)
Electricity:	Total global energy output of electricity in Joules. Electricity can be fully converted into other forms of energy; therefore, its exergy content is equal to its energy content.	(IEA, 2017)
Feedstock:	Organic fuels such as natural gas, or wood pulp used as a raw material for conversion processes. Global flows of feedstock are listed in terms of their LHV, which is converted to exergy.	(IEA, 2017)
Heat:	Heat flows from industrial processes that are used in other stages of the global supply chain. The energy of heat flows is listed by the IEA but not temperature. We use 600 °C for steel and chemical and petrochemicals and 180 °C for all other industries as is common in societal exergy analyses (Brockway et al., 2014; Serreno et al., 2014).	(IEA, 2017)
Losses:	Losses are computed by performing an exergy balance at each node of the diagram. They therefore include both exergy destruction and exergy loss due to waste products and heat loss.	Calculated
Non‐ferrous products:	Metal products that do not contain a dominant fraction of iron. Due to data restrictions, this study only addresses data for aluminium, copper, and zinc, the three major non‐ferrous metals produced worldwide.	(World Aluminium, 2017) (International Copper Study Group, 2015) (International Lead and Zinc Study Group, 2018)
Non‐metallic products	This includes all mineral products with no metallic content such as cement and glass.	(USGS, 2017) (Glass For Europe, 2015)
Oil products:	Oil‐based products that are not used as feedstock, such as kerosene or bitumen. Listed by LHVs which are converted.	(IEA, 2017)
Paper products:	This includes both paper and paperboard products.	(FAO, 2018)
Plastics:	This includes thermosets, thermoplastics, and fibers.	(Levi & Cullen, 2018)
Primary energy:	Refers to unprocessed fuels, such as biomass, coal, or crude oil. Extraction data is listed as Lower Heating Values.	(IEA, 2017)
Raw materials:	Includes all materials used as the base products (not Feedstock) for conversion processes. This includes biomass, scrap, and minerals.	(IEA, 2017) (Statista, 2017) (USGS, 2017)
Steel products:		(World Steel Association, 2017)
Uranium ore:	Unprocessed uranium ore worldwide. Exergy is calculated by summing uranium’s chemical and nuclear exergy.	(World Nuclear Association, 2017)

Industries are complex systems which feature interconnected flows, which make it challenging to collect and plot global data in Sankey diagrams. Previous studies have generated Sankey diagrams of exergy flows within an industrial site (Michalakakis et al., 2019) or for a given sector (Gonzalez Hernandez et al., 2018), but extending this same level of detail to a global scale would make the diagrams overly complex. For clarity, industries included in the global exergy map model are simplified to a set of inputs and outputs.

Inefficiencies at each stage of the Sankey diagram are computed by an exergy balance at each node, consisting of subtracting useful outputs from the sum of all exergy inputs. Therefore, the "Losses" flow includes both exergy destruction and exergy loss through waste products and heat loss.

Exergy literature distinguishes between efficiency definitions that compare the useful output to the total input and definitions that compare the desired product or result to the fuel and resource expended to achieve that result, developed in the 1990s (Bejan et al., 1995; Tsatsaronis, 1993). The latter definition deals with cases where a high amount of exergy flows through a system untransformed, which artificially inflates the efficiency value and requires detailed knowledge of the purpose and function of the system to accurately determine the product and fuel terms (Nguyen et al., 2014). As is common in societal exergy analyses however, we adopt an input‐output definition (Brockway et al., 2014; Sousa, et al., 2017). This allows for the consistent comparison of different sectors as data required to calculate a fuel‐product efficiency definition are not available for most sectors in this study.

## RESULTS

This section outlines the outputs of the exergy calculator and compares computed exergy values of selected substances to literature values. The results of the global resource exergy analysis are then presented, with a further analysis of the refining, utilities, and industrial sectors.

### Exergy database and calculator

The exergy calculator is able to return exergy conversion values for 860 individual chemical species, with 1480 separate entries overall, as well as all the possible user‐devised homogenous mixtures of up to eight chemical elements. For organic materials, where chemical data as well as formulae are both empirical, the standard deviation of results for a given entry never exceeds 5%. Table 3 lists exergy conversion factors for some relevant substances used for the global exergy map, to provide an example of the types of values returned. These are compared to similar results obtained in other academic studies, shown under "Reference" in Table 3.

**TABLE 3:** Selection of calculator outputs and comparison with analogous values obtained in other studies

Material	Calculated exergy	Reference exergy	Relative difference	Reference
*Inorganic materials*				
Aggregate	500 kJ/kg	620 kJ/kg	16%	(Koroneos et al., 2012)
Aluminum	29,000 kJ/kg	32,900 kJ/kg	12%	(Kotas, 1995)
Cement	1100 kJ/kg	1000 kJ/kg	10%	(Ari, 2011)
Copper	2100 kJ/kg	2100 kJ/kg	0%	(Kotas, 1995)
Limestone	60 kJ/kg	60 kJ/kg	0%	(Morris & Szargut, 1986)
Steel	7100 kJ/kg	6800 kJ/kg	4%	(Szargut, 2005)
Zinc	5400 kJ/kg	5400 kJ/kg	0%	(Kotas, 1995)
*Organic materials*				
General biomass	21,000 kJ/kg	20,200 kJ/kg	4%	(Song et al., 2011)
Paper	17,000 kJ/kg	20,200 kJ/kg	16%	(Song et al., 2011)
Thermoplastics	35,000 kJ/kg	38,900 kJ/kg	10%	(Eboh et al., 2016)
Thermosets	23,000 kJ/kg	20,800 kJ/kg	9%	(Eboh et al., 2016)
Wood	20,500 kJ/kg	20,800 kJ/kg	1%	(Song et al., 2011)
	1.04 J/J LHV	1.11 J/J LHV	6%	(Kotas, 1995)
*Organic fuels*				
Coal	1.01 J/J LHV	1.04 J/J LHV	3%	(Kotas, 1995)
Crude oil	46,600 kJ/kg	44,800 kJ/kg	4%	(Kotas, 1995)
	1.02 J/J LHV	1.06 J/J LHV	4%	(Kotas, 1995)
Natural gas	1.02 J/J LHV	1.04 J/J LHV	2%	(Kotas, 1995)
Oil products	1.04 J/J LHV	1.06 J/J LHV	2%	(Kotas, 1995)

It can be seen from this table that results from the calculator generally converge significantly with results obtained by previous studies. Discrepancies are largely below 5%. These literature values are indicative and a wide range of values can be found in previous works. Factors such as homogeneity assumptions for instance, can affect the accuracy of the results for materials such as heat‐treated steels.

For other raw inorganic materials such as aggregate (Koroneos et al., 2012), discrepancies in exergy may be due to differing chemical compositions in the calculations. For example, the chemical composition and microstructure of locally sourced construction materials such as aggregate rock, may vary widely between locations. Finally, differences with reference values for organic material may result from statistical variations as the calculator output is derived from an average of different correlations. This effect, however, is minimal and so is neglected within this study.

### Mapping global exergy flows

**FIGURE 2:**
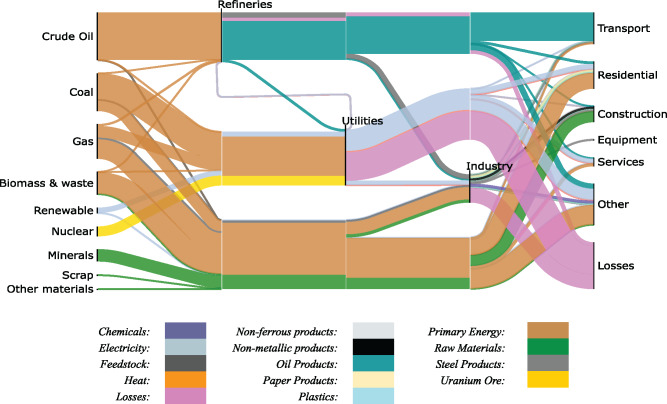
Sankey diagram of global exergy flows in 2013 from extraction to end use. White lines do not signify a process but rather act as waypoints for flow mixing and separation. Underlying data used to create this figure can be found in Supporting Information S2

Figure 2 shows the global Sankey diagram of material and energy flows for 2013, tracking the transformation of exergy from natural resources (left) to end‐use (right). The underlying data can be found in Supporting Information S2. From the diagram, we calculate a global primary to final exergy conversion efficiency of approximately 72%. Older studies for primary‐to‐final energy conversion have come up with similar results (Nakicenovic et al., 1996), while more recent works that include the end‐use stage (not included in this work) have found efficiencies of 10–15% (Brockway et al., 2014). Therefore, the world is theoretically capable of avoiding exergy losses and destruction of approximately 170 EJ annually in the upstream conversion of resources. Further savings are also available in downstream sections of the material supply chain.

A separate set of Sankey diagrams (for the three main conversion stages of refining, utilities and industry) are presented below, for comparison with existing studies, to validate the exergy method and to provide more detailed insight. Underlying data for all the following diagrams are provided in the Supporting Information S1 and S2 for this study.

### Conversion processes in detail

**FIGURE 3:**
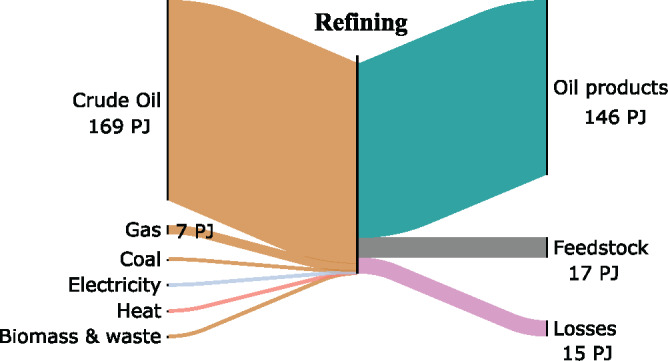
Sankey diagram of exergy flows at the Refining stage. Underlying data used to create this figure can be found in Supporting Information S1

Figure 3 shows the “refineries” node, where electricity, heat, and coal are used to provide energy to the process, whereas the crude oil input is split feedstock, for oil products, and process energy. The underlying data can be found in Supporting Information S1. The main output from refineries is oil products (i.e., chemicals used to make plastic and rubber products). A small proportion of the output is used as feedstocks to other sectors, lubricants or paraffins, and oil products primarily used in transport (diesel, kerosene, jet fuel) and construction (bitumen).

The overall Second‐Law efficiency of the refinery sector is 92%, slightly below the First‐Law efficiency of 98% deduced by the International Energy Agency (IEA, 2015a) . This high efficiency value is explained in part due to the use of the input‐output efficiency definition which fails to take into account the complex transformations taking place in the sector, for example, transiting exergy. Another factor underpinning this and, mostly, the high energy efficiency is that the refining sector has historically pursued energy and material efficiency as a means of improving profitability in a competitive environment (Han et al., 2015). Further improvement efforts are likely to yield marginal returns in energy, emission, and cost savings.

**FIGURE 4:**
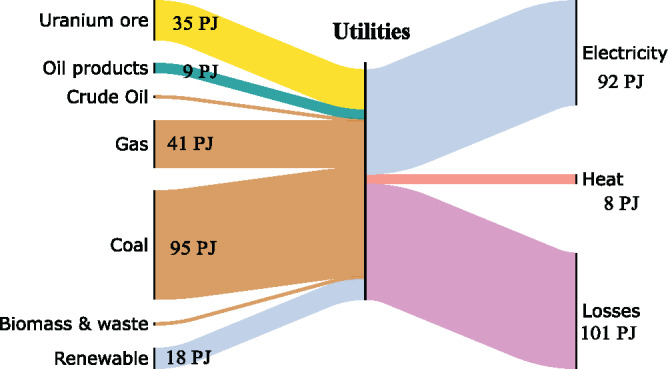
Sankey diagram of exergy flows at the Utilities stage. Underlying data used to create this figure can be found in Supporting Information S1

The "Utilities" stage (Figure 4) represents global power generation in 2013, including conventional fossil fuel generation as well as biomass, nuclear power, and renewables (IEA, 2015a,b,c). The underlying data can be found in Supporting Information S1. The outputs of electrical exergy shown in this diagram reflect actual power station output rather than final consumption, which neglects potentially large losses caused by electricity transmission and distribution. These are estimated at 8.5% of electricity output globally (IEA, 2017).

Renewable electricity in this figure is included in the electricity output but also shown as an input in the Utilities, even though it is not a primary fuel like gas and coal. As there is no physical material input required for the generation of renewable electricity, this convention is used to avoid the impression that renewable electricity is produced from nothing. This, however, affects calculation of exergy efficiency as renewable generation effectively acts as a 100% efficient means of generating power, significantly raising the overall performance of this stage. Indeed, the exergy efficiency of 50% of the utilities sector drops to 44% if the renewable generation is excluded, while the IEA estimates a global energy efficiency value of 41% (IEA, 2015b). Power generation is a significant cause of loss in the upstream global supply chain, with exergy losses and destruction of about 100 EJ per year. Combined with the sector’s relatively low exergy efficiency of 44%, this highlights the potential for targeting efficiency improvements in power generation. Indeed, a recent review of exergy analyses on power plants found that coal plants operate at below 35% exergy efficiency while gas and combined cycle power plants operate at above 50% (Ahmadi et al., 2019). This highlights the negative impact of the presence of coal plants in the global power sector, decreasing the overall exergy efficiency.

**FIGURE 5:**
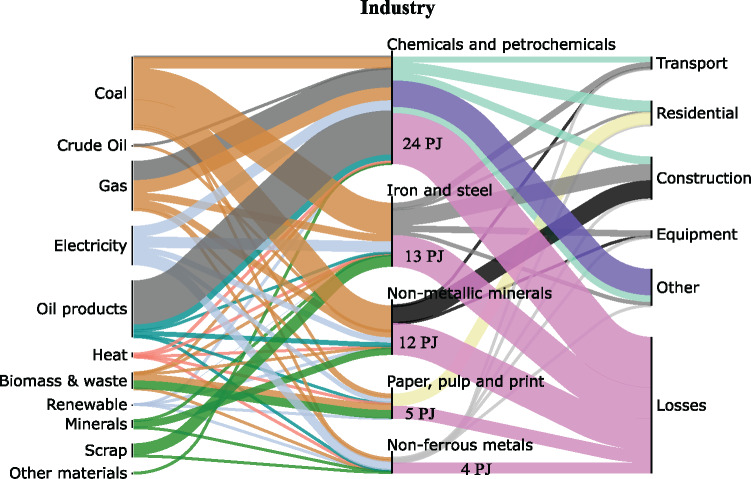
Sankey diagram of exergy flows at the Industry stage. Underlying data used to create this figure can be found in Supporting Information S1

Figure 5 depicts the Sankey diagram of the industrial sector and in particular, the five largest industries: ferrous and non‐ferrous metals, chemicals, minerals, and paper manufacturing. The underlying data can be found in Supporting Information S1. These industries had a combined exergy conversion efficiency of approximately 50% in 2013 and account for about 68% of industrial energy use (Luis & Van Der Bruggen, 2014), with other industrial sectors being included in the "Other" node at the "End‐Use" stage.

Inputs to the chemical and petrochemical sector consist mainly of energy and petrochemical feedstock, such as natural gas which is used for fertilizer production (IEA, 2017), while outputs include various types of plastics, to chemical fertilizers, and other chemical substances (Levi & Cullen, 2018). The dominant share of inputs (58.6%) originates from petrochemical feedstock, rather than energy, which explains why Chemicals and Petrochemicals appear to dominate other industrial sectors.

Figure 5 indicates that chemical and petrochemicals production is about 53% efficient, iron and steel sector is about 50% efficient, when measured in exergy. Recent studies focusing on the steel sector have yielded results close to ours at around 45% (Wu et al., 2016), or lower at 33% (Gonzalez Hernandez et al., 2018). The discrepancy could be explained by some of the material by‐products not being considered valuable in more specialized studies, which leads to decreased efficiency values. In addition, the second source is an average of all global steel‐making and therefore has a different boundary definition. The overall efficiency of non‐metallic minerals processing is 36%, which is comfortably located within the range of efficiencies found by previous studies (Madlool et al., 2012), 18% to 49%, for typical cement plants. The exergy efficiency of the paper manufacturing sector is calculated as 49% similar to other literature results for a typical paper mill (Al‐Ghandoor et al., 2010; Utlu & Kincay, 2013). The resource efficiency of the non‐ferrous metals sector is about 31%, which is similar the exergy efficiency of primary aluminium manufacturing (which accounts for 93% of non‐ferrous sector exergy output), lies between 15.9% and 39.9% (Balomenos et al. 2011).

## DISCUSSION AND CONCLUSION

This section discusses some of insights and conclusions obtained from the calculator and the potential for improving it, followed by a discussion of the conclusions from the exergy mapping case study and ends with an outline of potential improvements to the mapping study.

### Exergy calculator

The aim of this project was to create an exergy calculation tool that allows users to easily compute the chemical exergy of a wide range of substances to study global flows of exergy. This work addresses the lack of any comprehensive datasets for material exergy in current literature, which causes large scale exergy analyses to be computationally expensive. To fill this gap, this project focused on the creation of an exergy calculator which could be used for such studies and combined a range of different chemical exergy calculation approaches for a variety of material types into one database. These materials include organic, inorganic, and even user‐defined substances.

The calculator designed and implemented in this project now returns reliable values for 1480 substances, considerably simplifying the exergy study of large and complex systems. Where a given material can take a variety of different forms (e.g., steel alloy), the calculator is structured to return results that will give the user a range of different exergy values so the user can pick the most appropriate for their application. Inorganic materials, with their inherent complexity and uncertainty, are dealt with by providing mean values for chemical exergy and standard deviations which allow the user to obtain a sense of the uncertainty in the exergy values they are using for their application.

Currently, the calculator only returns values of chemical exergy, which is appropriate considering the scope of this project. However, users may find it useful to be able to compute other forms of exergy, such as physical, kinetic, or potential exergy. The calculator could therefore be updated to include input fields for temperature, pressure, height, or speed. This will require additional relevant values to the database, such as heat capacities of individual materials.

The calculator also omits nuclear exergy, equal to energy liberated during fission of fissile materials, which may be a considerable oversight given that nuclear power generation represents approximately 11% of worldwide electricity generation (EIA, 2017). For example, the chemical exergy of uranium ore returned by the calculator is about 7.1 MJ/kg, whereas its corresponding nuclear exergy would be 40,000 times larger at 292,000 MJ/kg (Szargut, 2005). Therefore, for the purpose of this project, nuclear exergy was calculated separately and added to the chemical exergy for relevant materials included in the Sankey diagram, such as uranium ore. In the long term, however, a functionality to calculate nuclear exergy would be a useful addition to the calculator.

### Resource mapping

Subsequently, this study exemplified how the calculator could be used to develop a diagram of global exergy flows as a proof of concept. Outputs from this tool were used to produce an example of a global Sankey diagram of exergy‐based resource flows. We validated our work with corresponding literature results and confirmed that the obtained diagrams closely matched previous results, demonstrating that the calculator could be a useful tool for this kind of analyses.

The global Sankey diagram highlighted some of most inefficient the global sectors and returned their second‐law efficiency. This was useful to gauge the opportunities for efficiency improvement and material demand reduction. However, the use of the input–output efficiency definition, rather than a product–fuel definition for instance, may affect the efficiency values for the sectors, as in the case of the refining sector.

These findings will allow policymakers to effectively target the sectors featuring the largest potential for progress in resource‐intensity. For example, power generation and chemicals manufacturing were responsible for 63% and 12% of global exergy losses and destruction, respectively, while simultaneously only using 41% and 33% of the available energy they consumed. This therefore reveals that effective climate policy should prioritize addressing efficiency issues in these two sectors as they are most likely to have a significant impact on the resource intensity of the global economy.

We believe the calculator developed in this project is a relevant and important tool which can facilitate further studies in exergy mapping of resource efficiency at a wide range of scales, from the process scale to the global scale, particularly where chemical exergy dominates the exergy flows. This, in turn, provides a firm technical tool for guiding policy that targets resource efficiency by clearly and pragmatically representing the greatest potential candidate sectors for improvement. The calculator has already been presented at the 3rd International Exergy Economics workshop in Lisbon (Cullen, 2018) as a helpful tool in accelerating the pace of exergy mapping.

### Potential improvements

Because of the amount of data to be modeled, and the difficulty in acquiring extensive information from a range of different industrial sectors, the Sankey diagram had to be kept at a relatively low level of detail. As a result, much of the information displayed can be misleading if not carefully considered. For example, because the industrial sectors represented only amount to 68% of all industrial energy consumption (Luis & Van Der Bruggen, 2014), it is difficult to draw any meaningful conclusion about the overall exergy consumption and efficiency of the sector as a whole. It does provide useful insight, however, when comparing the relative size of exergy losses and destruction between individual sectors.

Additionally, losses and destruction in this study are addressed by making a second‐law exergy balance at each node (instead of evaluating losses at each process within the node itself), which may point to misleading conclusions. This is because outputs are divided between useful exergy output, such as crude steel for the iron and steel sector, and losses. Indeed, the arbitrary selection of these "useful" products can greatly affect efficiency results; for instance, steel slag in this study is not taken as a useful output (therefore being included as a ‘Loss’), whereas it is conventionally recycled for use as an aggregate material in construction.

To address this issue, it may be necessary to take a more detailed approach to each stage, much like what was accomplished by previous studies for the steel industry by breaking down the sector into its component stages, generating separate exergy balances, and adopting a rigorous approach to handling outputs (Gonzalez Hernandez et al., 2018). Considering the scope of this project, however, this approach would be unnecessarily complex, which is why the simplified balance method used here is considered sufficient to produce an approximate overview of exergy flows.

The current global map is broken down into vertical slices: energy conversion, industrial production, and end use. These slices do not include some other activities such as manufacturing which are outside the scope of this study. For example, the manufacturing stage where materials are shaped and combined into final goods is not displayed. Even though including a "manufacturing" node would not reflect the change of embodied exergy of materials as they are shaped (chemical exergy ignores all factors other than a material’s composition), this would provide insight into exergy lost in the manufacturing processes. However, this was not considered within the scope of this project as mapping flows of exergy between different manufacturing operations is prohibitively arduous and would only reflect minimal exergy consumption in comparison to other economic sectors shown in the global diagram.

The last vertical slice of the Sankey diagram corresponds to the end uses of exergy. However, a distinction must be made between "Final Exergy" (the input of exergy to the last stage of use such as lighting) displayed in this diagram, and "Useful exergy" (Cullen & Allwood, 2010)—the useful exergy output of the last stage of use, such as the light intensity; these values are rarely equal due to imperfect conversion methods (e.g., lightbulbs) of end use. Indeed, previous research (Nakicenovic et al., 1996) determined that average global conversion efficiency from final output to end‐use was of the order of 15%. Therefore, an additional stage, denoted as "Useful Exergy" should be added to the present diagram to address this issue; this would be dealt with by evaluating the average exergy efficiency of the multiple stages of conversion from final to useful exergy, much like what was accomplished by previous studies.

## Supplementary Information

**Supporting Information S1**: This supporting information S1 provides data used in the construction of the Sankey diagrams presented in the manuscript (Figures 3, 4, and 5).

**Supporting Information S2**: This supporting information S2 provides the data used to create Figure 2 in the main text.

**Supporting Information S3**: This supporting information S3 provides the raw material flow and chemical energy conversion data.

## Footnotes

1 https://www.refficiency.org/projects/exergy‐calculator/
