# Supplementary material for: Calculating the chemical exergy of materials
Source: J Ind Ecol. 2021 Mar 31;25(2):274–87. doi: 10.1111/jiec.13120 (PMC13079481; doi:10.1111/jiec.13120)
Supplement: Supplementary file 1 — Supporting Information S1: This supporting information S1 provides data used in the construction of the Sankey diagrams presented in the manuscript (Figures 3, 4, and 5). [file 44498_2021_2502003_MOESM1_ESM.pdf]

SUPPORTING INFORMATION FOR:

Michalakakis, C., J. Fouillou, A. Gonzalez Hernandez, and J.M. Cullen. 2021. Calculating the Chemical Exergy of Materials. *Journal of Industrial Ecology.*

Summary

This supporting information S1 provides data used in the construction of the Sankey diagrams presented in the manuscript (Figures 3, 4, and 5).

Table S1-1: Data plotted in Figure 3 of the main text

Inputs			Outputs		
	Exergy (PJ)	*% of total*		*Exergy (PJ)*	*% of total*
Crude Oil	169,000	95.6	*Oil products*	145,600	82.1
Gas	7,060	4.0	*Feedstock*	16,800	9.5
Coal	30	0.02	*Losses*	14,900	8.4
Electricity	217	0.1			
Heat	400	0.2			
Biomass & waste	10	0.01			
Total	176,700	100.0	*Total*	176,700	100.0

Table S1-2: Data plotted in Figure 4 of the main text

Inputs			Outputs		
	Exergy (PJ)	*% of total*		*Exergy (PJ)*	*% of total*
Coal	95,300	47.4	*Electricity*	92,000	45.7
Gas	41,000	20.3	*Heat*	4,400	2.2
Uranium	34,630	17.2	*Losses*	104,900	52.1
Renewables	18,060	9.0			
Oil Products	8,620	4.3			
Biomass & waste	2,100	1.0			
Crude Oil	1,600	0.8			
Total	201,300	100.0	*Total*	201,300	100.0

Table S1-3: Data plotted in the Chemicals and Petrochemicals section in Figure 5 of the main text.

Inputs			Outputs		
	Exergy (PJ)	% of total		Exergy (PJ)	% of total
Oil Products (Feedstock)	16,800	41.3	*Thermoplastics*	7,920	19.5
Oil Products (Energy)	2,400	5.9	*Thermosets, fibre & elastomer*	3,600	8.9
Crude Oil (Feedstock)	80	0.2	*Ammonia fertilizers*	5,500	13.5
Gas (Feedstock)	7,200	17.7	*Solvents, additives & explosives*	450	1.1
Gas (Energy)	5,000	12.3	*Losses*	23,200	57.0
Coal (Energy)	4,200	10.3			
Electricity	4,100	10.1			
Heat	550	1.4			
Biomass & waste	70	0.2			
Renewables	0	0.0			
Other	270	0.7			
Total	40,670	100.0	*Total*	40,670	100.0

Table S1-4: Data plotted in the Iron and Steel section in Figure 5 of the main text.

Inputs			Outputs		
	Value (PJ)	*% of total*		*Value (PJ)*	*% of total*
Coal	12,200	49.5	*Steel Products*	12,500	50.6
Gas	2,770	11.2	*Losses*	12,200	49.4
Electricity	4,150	16.8			
Oil products	390	1.6			
Heat	343	1.4			
Biomass & waste	160	0.6			
Minerals	240	1.0			
Scrap	4,410	17.9			
Total	24,700	100.0	*Total*	24,700	100.0

Table S1-5: Data plotted in the non-metallic minerals section in Figure 5 of the main text.

Inputs			Outputs		
	Exergy (PJ)	*% of total*		*Exergy (PJ)*	*% of total*
Coal	9,790	51.1	*Cement*	6,800	35.5
Crude Oil	300	1.6	*Glass*	100	0.5
Gas	2,300	12.0	*Losses*	12,249	64.0
Electricity	2,100	11.0			
Oil Products	1,700	8.9			
Heat	29	0.3			
Biomass & waste	350	1.8			
Renewables	0	0.0			
Limestone	2,500	13.0			
Sand and Gravel	80	0.4			
Total	19,149	100.0	*Total*	19,149	100.0

Table S1-6: Data plotted in the paper and pulp section in Figure 5 of the main text.

Inputs			Outputs		
	Exergy (PJ)	*% of total*		*Exergy (PJ)*	*% of total*
Coal	910	9.5	***Paper products***	4,700	49.8
Gas	990	10.4	***Losses***	4,730	50.2
Electricity	1,520	15.9			
Oil Products	220	2.3			
Heat	130	2.5			
Biomass (Energy)	2,500	26.2			
Biomass (Feedstock)	3,150	33.0			
Renewables	10	0.1			
Total	9,430	100.0	***Total***	9,430	100.0

Table S1-7: Data plotted in the non-ferrous metals section in Figure 5 of the main text.

Inputs			Outputs		
	Exergy (PJ)	*% of total*		*Exergy (PJ)*	*% of total*
Coal	920	15.0	***Aluminium Products***	1,890	30.8
Gas	680	11.1	***Copper Products***	70	1.1
Electricity	3,230	52.6	***Zinc Products***	70	1.1
Oil products	270	4.4	***Losses***	4,109	66.9
Heat	39	0.6			
Biomass & waste	10	0.2			
Renewables	10	0.2			
Minerals	120	2.0			
Scrap	860	14.0			
Total	6,139	100.0	***Total***	6,139	100

Note: Data for Figure 2 in the main text are simply the summation of the data provided in the tables above.
